# Salicylic Acid and *N*-Hydroxypipecolic Acid at the Fulcrum of the Plant Immunity-Growth Equilibrium

**DOI:** 10.3389/fpls.2022.841688

**Published:** 2022-03-10

**Authors:** Alyssa Shields, Vanessa Shivnauth, Christian Danve M. Castroverde

**Affiliations:** Department of Biology, Wilfrid Laurier University, Waterloo, ON, Canada

**Keywords:** salicylic acid, *N*-hydroxypipecolic acid, pipecolic acid, plant immunity, plant growth, plant development, plant hormone, growth-defense tradeoff

## Abstract

Salicylic acid (SA) and *N*-hydroxypipecolic acid (NHP) are two central plant immune signals involved in both resistance at local sites of pathogen infection (basal resistance) and at distal uninfected sites after primary infection (systemic acquired resistance). Major discoveries and advances have led to deeper understanding of their biosynthesis and signaling during plant defense responses. In addition to their well-defined roles in immunity, recent research is emerging on their direct mechanistic impacts on plant growth and development. In this review, we will first provide an overview of how SA and NHP regulate local and systemic immune responses in plants. We will emphasize how these two signals are mutually potentiated and are convergent on multiple aspects—from biosynthesis to homeostasis, and from signaling to gene expression and phenotypic responses. We will then highlight how SA and NHP are emerging to be crucial regulators of the growth-defense balance, showcasing recent multi-faceted studies on their metabolism, receptor signaling and direct growth/development-related host targets. Overall, this article reflects current advances and provides future outlooks on SA/NHP biology and their functional significance as central signals for plant immunity and growth. Because global climate change will increasingly influence plant health and resilience, it is paramount to fundamentally understand how these two tightly linked plant signals are at the nexus of the growth-defense balance.

## Introduction

Plants rely on their two-tiered and interlinked innate immune system to initiate local responses against pathogenic attack (Jones and Dangl, 2006; Kim and Castroverde, 2020; Zhou and Zhang, 2020; Yuan et al., 2021). First, pattern-triggered immunity (PTI) is initiated after activation of cell surface pattern recognition receptors (PRRs) that typically recognize conserved pathogen-associated molecular patterns (PAMPs; Macho and Zipfel, 2014; Li et al., 2016; DeFalco and Zipfel, 2021). Second, a more robust effector-triggered immunity (ETI) is activated when pathogen effectors are recognized by intracellular nucleotide-binding leucine-rich repeat receptors (NLRs), often resulting in localized cell death (Zebell and Dong, 2015; Saur et al., 2021). Sustained immune activation at the local infection site primes unaffected systemic tissues against future biotic stress *via* systemic acquired resistance (SAR; Vlot et al., 2021; Zeier, 2021). Several key SAR inducers have been identified, including salicylic acid (SA), methyl SA, azelaic acid (AzA), glycerol-3-phosphate (G3P), dehydroabietinal (DA), nitric oxide (NO), reactive oxygen species (ROS), pipecolic acid (Pip), and *N*-hydroxypipecolic acid (NHP; Wendehenne et al., 2014; Singh et al., 2017; Hartmann et al., 2018).

A central regulator of local and systemic immunity is the plant hormone SA (Zhang and Li, 2019). Because it serves various roles, SA levels and metabolism are altered during immune responses to suit the plant’s needs (Dempsey et al., 2011). SA is produced *via* two independent pathways: isochorismate synthase (ICS) and phenylalanine ammonia lyase (PAL) pathways (Dempsey et al., 2011; Hartmann and Zeier, 2019; Zhang and Li, 2019; Huang et al., 2020a). In *Arabidopsis*, most of the pathogen-induced SA is produced through the ICS pathway involving pathogen-induced genes *ISOCHORISMATE SYNTHASE 1* (*ICS1*), *ENHANCED DISEASE SUSCEPTIBILITY 5* (*EDS5*), and *AVRPPHB SUSCEPTIBLE 3* (*PBS3*; Chen et al., 2009; Huang et al., 2020a). Of the two *Arabidopsis* ICS paralogs, ICS1 plays a major role in SA synthesis following infection (Nawrath and Metraux, 1999; Wildermuth et al., 2001; Garcion et al., 2008). In plastids, ICS1 converts chorismate to isochorismate, which is transported by EDS5 to the cytosol (Garcion et al., 2008). PBS3 and EPS1 then catalyze the final conversions to SA (Rekhter et al., 2019; Torrens-Spence et al., 2019). Although low SA levels can be transported to systemic tissues during SAR, its long-distance mobility alone is not responsible for SAR establishment (Vernooij et al., 1994; Lim et al., 2020). It is proposed that SA contributes to systemic propagation of defenses alongside other signaling molecules (Lim et al., 2020; Vlot et al., 2021).

Another metabolite involved in plant immunity is NHP, a hydroxylated derivative of the non-protein amino acid Pip that can induce SA accumulation (Návarová et al., 2012; Hartmann et al., 2018; Wang et al., 2018). The NHP biosynthetic pathway is inducible by pathogens and leads to SAR (Hartmann et al., 2018). NHP can induce defense gene expression, amplify the resistance response, synergistically function with SA, and promote the hypersensitive response (Hartmann et al., 2018). Recent exciting studies have provided detailed insights into NHP biosynthesis and mobilization. Three pathogen-inducible genes are involved in NHP biosynthesis: *AGD2-LIKE DEFENSE RESPONSE PROTEIN 1* (*ALD1*), *SAR DEFICIENT 4* (*SARD4*), and *FLAVIN-DEPENDENT MONOOXYGENASE 1* (*FMO1*; Hartmann and Zeier, 2018). ALD1 is an L-Lys-α-aminotransferase that deaminates L-Lys, spontaneously leading to dehydropipecolic acid intermediates (Hartmann and Zeier, 2018). These are reduced by SARD4 to Pip, which is then converted by FMO1 to NHP (Hartmann and Zeier, 2018). The local and systemic accumulation of Pip and NHP after pathogen attack are necessary for SAR (Hartmann and Zeier, 2018).

Deployment of SA, NHP, and other defense responses must be balanced with the plants’ ability to grow and/or develop in order to optimize overall fitness (Huot et al., 2014). This “growth-defense equilibrium” paradigm has been postulated due to limited resources that must be balanced leading to reciprocal tradeoffs (Coley et al., 1985). Alternatively, this is due to interlinked and conditional coordination between growth and immune responses depending on the environment (Kliebenstein, 2016). In terms of SA and NHP, over-accumulating mutants exhibit decreased growth (Abreu and Munné-Bosch, 2009; Pastorczyk-Szlenkier and Bednarek, 2021), reflecting that SA/NHP mediate the delicate equilibrium between plant growth and immunity.

## Convergence of SA and NHP Biosynthesis and Signaling

**Figure 1:**
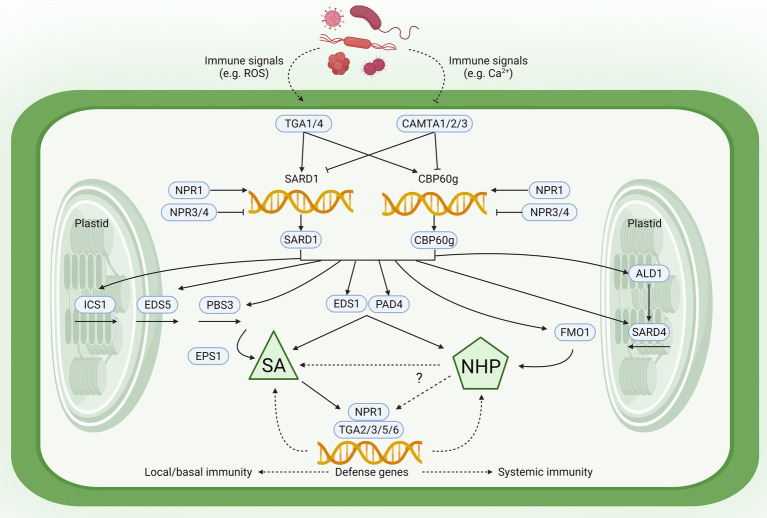
Regulatory convergence and mutual potentiation of salicylic acid (SA) and *N*-hydroxypipecolic acid (NHP) biosynthesis and signaling. Upstream immunity-associated signals [e.g., reactive oxygen species (ROS), Ca^2+^] lead to activation/repression of TGACG SEQUENCE-SPECIFIC BINDING PROTEIN 1 (TGA1)/4 transcriptional activators and CALMODULIN-BINDING TRANSCRIPTION ACTIVATOR (CAMTA) transcriptional repressors. Along with the antagonistic SA receptors NONEXPRESSER OF PR GENES 1 (NPR1; co-activator) and NPR3/4 (co-repressors), TGA1/4 and CAMTA1/2/3 control expression of CALMODULIN-BINDING PROTEIN 60-LIKE G (*CBP60g*) and SAR DEFICIENT 1 (*SARD1*) that encode functionally redundant master transcription factors of plant immunity. SARD1 and CBP60g directly bind the promoters of SA biosynthetic (*ICS1*, *EDS5*, and *PBS3*) and NHP biosynthetic genes (*ALD1*, *SARD4*, and *FMO1*). Central immune regulators ENHANCED DISEASE SUSCEPTIBILITY 1 (EDS1) and PHYTOALEXIN DEFICIENT 4 (PAD4; which mediate both pattern-triggered immunity and effector-triggered immunity) are also required for SA and NHP accumulation. Downstream of their biosynthesis, SA directly activates while NHP indirectly activates the SA receptor NPR1. NPR1 then promotes TGA-directed transcription of key defense genes for local/basal and systemic immune responses. Created with BioRender.com.

To understand the relationship between immunity and growth *via* the SA and NHP pathways, it is important to highlight the tight mechanistic linkage between these two central immune-activating metabolites (Figure 1; for detailed review, see Zeier, 2021). SA and NHP biosynthesis and downstream signaling are closely intertwined, relying on overlapping regulatory proteins and signaling components (Sun et al., 2015; Hartmann and Zeier, 2019; Ding and Ding, 2020). The SA pathway genes *ICS1*, *EDS5*, and *PBS3* and the NHP biosynthetic genes *ALD1*, *SARD4*, and *FMO1* are regulated *via* two partially redundant master transcription factors SAR DEFICIENT 1 (SARD1) and CALMODULIN-BINDING PROTEIN 60-LIKE G (CBP60g; Wang et al., 2011; Sun et al., 2015; Huang et al., 2020a). SARD1 and CBP60g activation by pathogen infection and/or immune elicitation leads to increased SA and NHP levels (Hartmann and Zeier, 2019; Huang et al., 2020a).

Full induction of *SARD1* and *CBP60g* gene expression requires TGACG SEQUENCE-SPECIFIC BINDING PROTEIN 1 and 4 (TGA1 and TGA4) transcription factors, which modulate SA and NHP levels (Hartmann et al., 2018; Sun et al., 2018; Zhang and Li, 2019). TGA1 and TGA4 are paralogs of the TGA transcription factor family, which specifically bind variants of the palindromic sequence TGACGTCA in target gene promoters (Xiang et al., 1997). In addition to TGA1/4, other TGAs include TGA2/3/5/6, which are essential for responses to SA and NHP (Kesarwani et al., 2007; Nair et al., 2021). Higher-order *tga* mutants have significantly reduced sensitivity to SA and NHP (Zhang et al., 2003; Nair et al., 2021), which could potentially explain their SAR-deficient phenotypes (Zhang et al., 2003; Kesarwani et al., 2007). The requirement of these TGAs for SA- and NHP-mediated transcriptional reprogramming is expected since TGAs recruit the master coactivator and SA receptor NONEXPRESSER OF PR GENES 1 (NPR1), which is required for SA- and NHP-responsive expression (Ding et al., 2018; Nair et al., 2021). In addition to TGAs, SA, and NHP biosynthesis and signaling can be modulated by CALMODULIN-BINDING TRANSCRIPTION ACTIVATOR (CAMTA) 1, 2, and 3—central transcriptional repressors in plant immunity that directly target *CBP60g* and *SARD1* promoters (Sun et al., 2020).

In addition to transcription factors, other proteins also control SA/NHP accumulation. These include two lipase-like proteins ENHANCED DISEASE SUSCEPTIBILITY 1 (EDS1) and PHYTOALEXIN DEFICIENT 4 (PAD4; Hartmann and Zeier, 2019; Zeier, 2021), which mediate both ETI and PTI. This potentially suggests the major importance of the SA and NHP pathways after immune activation. Interestingly, *EDS1* and *PAD4* are target genes of SARD1 and CBP60g (Sun et al., 2015), further reflecting the close mechanistic relationships of these immune regulators during SA/NHP production. Recent studies have identified another key component involved in local and systemic immunity—a Jumonji (JMJ) domain-containing H3K4 demethylase, JMJ14 (Li et al., 2020). In local leaves, JMJ14 positively regulates immunity by upregulating *ALD1*/*FMO1* transcription and enhanced SA-responsiveness; in distal leaves, JMJ14 is vital for systemic NHP accumulation and SAR (Li et al., 2020). The *jmj14* mutants exhibited reduced local and systemic defenses. Remarkably, JMJ14 positively regulates immunity-induced H3K4me3 histone enrichment in SA- and NHP-associated defense genes (Li et al., 2020). Altogether, these studies highlight the common and overlapping molecular players that impinge on the SA and NHP pathways.

## Mutual Potentiation of SA and NHP During Plant Immunity

Because of common overlapping SA and NHP regulators, it is not surprising that SA/NHP cooperatively and synergistically influence each other to induce SAR (Figure 1; for detailed review, see Zeier, 2021). This mutual amplification is best exemplified by their effect on each other’s biosynthetic genes. NHP biosynthetic enzymes ALD1 and FMO1 are required for systemic SA accumulation (Mishina and Zeier, 2006; Cecchini et al., 2015). Indeed, NHP treatment directly induces and also primes SA biosynthetic gene expression (*ICS1*, *EDS5*, and *PBS3*) and SA production, as elegantly demonstrated by Yildiz et al. (2021). Downstream of SA biosynthesis, NHP also primes SA-induced defense gene expression (Bernsdorff et al., 2016; Yildiz et al., 2021).

On the other hand, SA can enhance NHP-activated immunity and gene expression (Hartmann et al., 2018; Yildiz et al., 2021). In particular, both *ALD1* and *FMO1* gene expression can be directly upregulated by SA (Cecchini et al., 2015), although they also exhibit SA-independent expression (Bartsch et al., 2006; Bernsdorff et al., 2016). SA induction-deficient *sid2* mutants are SAR-deficient, but not to the same extent as NHP-deficient *ald1* and *fmo1* mutants (Hartmann et al., 2018; Yildiz et al., 2021). Potentially, this could be due to basal SA levels present in *sid2* mutants (Nair et al., 2021), but further genetic and molecular dissection is necessitated.

This mutual potentiation can be explained since SA- and NHP-mediated signaling both depend on the coactivator NPR1 (Návarová et al., 2012; Yildiz et al., 2021) and its paralogous corepressors NPR3 and NPR4, all of which can bind SA and regulate SAR (Fu et al., 2012; Wu et al., 2012; Fu and Dong, 2013; Ding et al., 2018; Liu et al., 2020). Both SA-induction of NHP biosynthetic genes and NHP-induction of SA-associated genes depend on the NPR1 regulatory module (Ding et al., 2018; Nair et al., 2021; Zeier, 2021). Overall, these demonstrate that SAR is dependent on mutual amplification of SA and NHP (Bernsdorff et al., 2016; Huang et al., 2020a; Nair et al., 2021; Yildiz et al., 2021), illustrating the cooperative interactions between these two central immune-activating metabolites.

## Mechanistic Impact of SA on Plant Growth and Development

**Figure 2:**
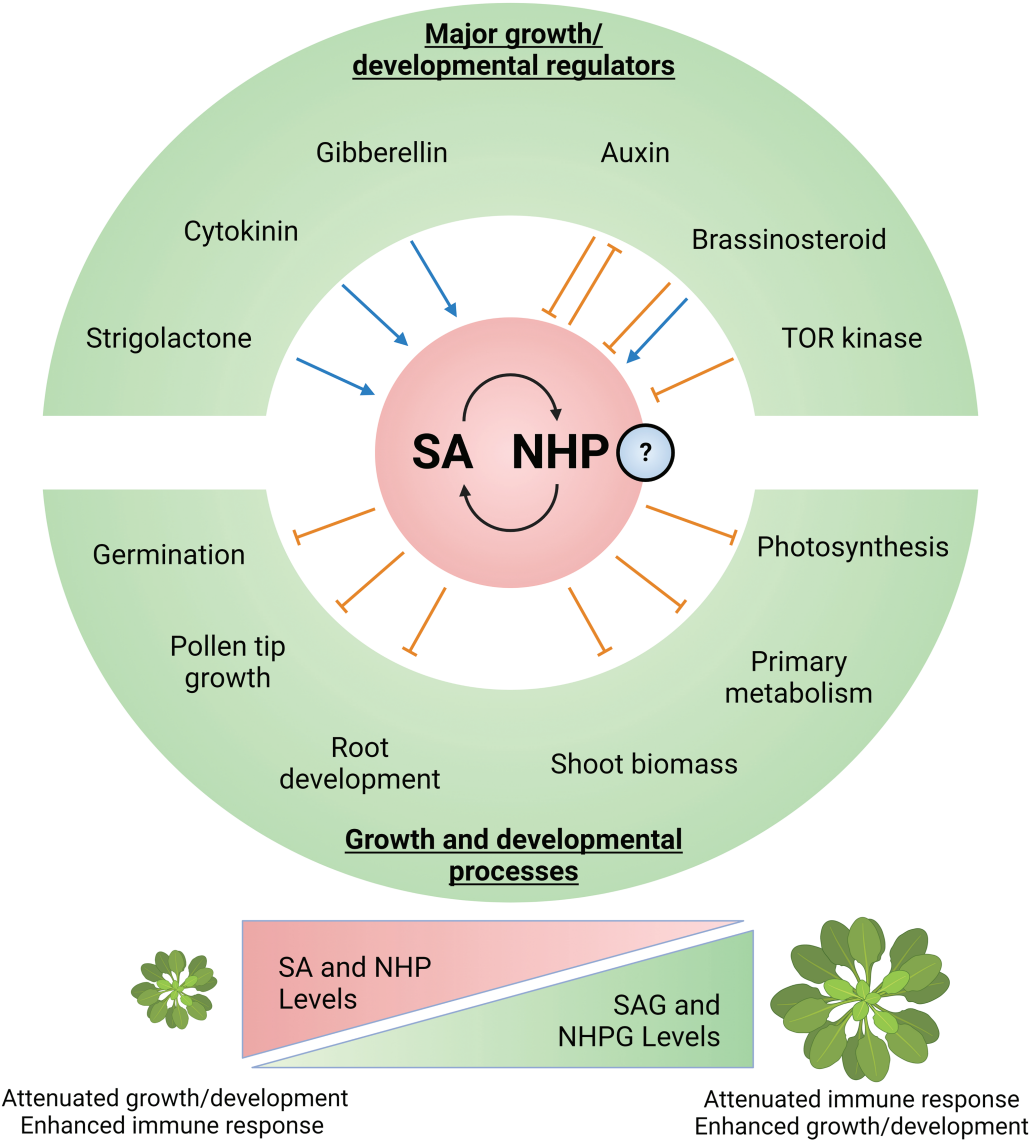
Salicylic acid and NHP at the nexus of the plant growth-defense balance. Major regulators of growth and development have synergistic and/or antagonistic relationships with SA and potentially with NHP. These include key plant hormones (auxin, brassinosteroid, gibberellin, cytokinin, and strigolactone) and the master regulatory kinase Target of Rapamycin (TOR). SA and potentially NHP could independently or synergistically impact various aspects of plant growth and development. In particular, SA has been shown to influence germination and apical hook development, pollen tip growth during floral development, root growth and patterning, shoot biomass accumulation, primary metabolism, and photosynthesis. Ultimately, levels and homeostasis between free bioactive SA/NHP and inactive storage forms (SAG/NHPG) allow plants to dynamically balance resources between growth and defense. Higher SA/NHP potentiates immune responses at the expense of growth, while lower SA/NHP promotes growth processes and modulates immunity. Created with BioRender.com.

Although SA is typically known as a defense hormone, it also affects plant growth and development (Figure 2) independently and/or *via* crosstalk with other hormones and signaling molecules (van Butselaar and Van den Ackerveken, 2020; Castroverde and Dina, 2021; Pokotylo et al., 2021; Saleem et al., 2021). SA-depleted *Arabidopsis* NahG transgenic plants are larger, while mutants with constitutively high SA levels such as *acd6–1* are dwarfed (Rivas-San Vicente and Plasencia, 2011). SA can also delay or inhibit seed germination in *Arabidopsis*, possibly from the resulting oxidative stress (Rajjou et al., 2006). This interplay between SA and ROS positively affects cell division in the quiescent center (QC), directly linking SA to root phenotypes (Wang et al., 2021). In agreement, SA-accumulating mutants and/or exogenous SA treatment can increase cell division in the QC by promoting ROS generation (Wang et al., 2021). Reproductive development is also modulated by SA. In *Arabidopsis*, SA inhibits pollen tube tip growth, whereas methylated SA promotes tip growth (Rong et al., 2016). The enzymes that interconvert between SA and MeSA (MeSA methylesterase and SA methyltransferase) can be found at the pollen tube apical regions, implying localized pollen tip synthesis (Rong et al., 2016). There is also an antagonistic effect between SA and ethylene-mediated apical hook formation, which is essential for growth above soil after germination (Huang et al., 2020b). Apical hooks are promoted by ethylene and involve transcription factors ETHYLENE INSENSITIVE 3 (EIN3) and ETHYLENE INSENSITIVE 3-like 1 (EIL1; Huang et al., 2020b). SA activates NPR1 and inhibits EIN3 binding to target gene promoters, such as *HLS1* (Huang et al., 2020b). Though varied, SA clearly has an impact on various growth and developmental processes, which are facilitated by the intricate crosstalk between SA and other signals (e.g., major growth hormones).

Auxin is important for growth and development (Lavy and Estelle, 2016); therefore, elucidating how SA impacts auxin biosynthesis/signaling is key to understanding the central role of SA in plant growth-defense balance. Since both SA and auxin biosynthetic pathways proceed from the precursor chorismate (product of the shikimate pathway), it is possible that one hormone shifts the shikimate pathway metabolic flux away from the other (Koo et al., 2020). SA can affect root meristem patterning, suggesting changes in auxin synthesis and transport (Pasternak et al., 2019). For example, exposure to low SA concentration (below 50 μM) promotes adventitious root formation in *Arabidopsis*, potentially by elevating root tip auxin levels to promote root meristem maturation (Pasternak et al., 2019). Because of this SA-auxin interplay, pathogens sometimes co-opt the auxin pathway to better infect plants (Pasternak et al., 2019). In response to pathogens, plants can use SA to repress the auxin pathway. SA can interact with and inhibit CATALASE2 (CAT2) to increase H_2_O_2_ levels, thereby repressing biosynthesis of the auxin precursor tryptophan by sulfenylating a key enzyme (Yuan et al., 2017). SA treatment also leads to increasing AUXIN RESISTANT/INDOLE-3-ACETIC ACID INDUCIBLE (Aux/IAA) repressor levels thereby repressing auxin-related gene transcription (Wang et al., 2007). In addition, SA can interfere with auxin transport by repressing clathrin-mediated endocytosis (Du et al., 2013). SA also antagonizes auxin by inhibiting protein phosphatase 2A resulting in auxin transporter PIN-FORMED 2 (PIN2) hyperphosphorylation, leading to attenuated root growth (Tan et al., 2020). Strikingly, SA can enhance adventitious root formation in cucumbers by competitively inhibiting the enzyme *Cucumis sativus* GRETCHEN HAGEN 3.5 (CsGH3.5), thereby increasing free auxin levels (Dong et al., 2020). Altogether, SA can influence aspects of plant growth and development by interfering with the auxin pathway.

Like auxins, gibberellins (GA) constitute another major class of hormones mediating growth and development (Emamverdian et al., 2020). During germination of the halophyte *Limonium bicolor* under salt stress, SA upregulated various genes involved in GA biosynthesis (Liu et al., 2019). Complementing this finding, exogenous GA increased expression of *NPR1* and *WRKY70*, resulting in elevated SA (Alonso-Ramírez et al., 2009).

## Mechanistic Impact of NHP on Plant Growth and Development

The impact of SA on growth and development is well-documented (Carviel et al., 2009; Rivas-San Vicente and Plasencia, 2011; Carella et al., 2014; van Butselaar and Van den Ackerveken, 2020; Pokotylo et al., 2021); however, the effect of NHP is only starting to be explored (Figure 2). For example, altering free NHP levels by inactivating UGT76B1-mediated glycosylation to NHPG can affect plant growth by decreasing rosette size and biomass (Bauer et al., 2021; Cai et al., 2021; Mohnike et al., 2021). Inhibited plant development and enhanced SAR was observed in the *ugt76b1* mutant, while overexpression led to opposite phenotypes (Bauer et al., 2021; Cai et al., 2021; Mohnike et al., 2021). Since NHP activates SAR, UGT76B1 dictates NHP levels and thus the SAR response (Bauer et al., 2021; Cai et al., 2021; Holmes et al., 2021; Mohnike et al., 2021). Interestingly, UGT76B1 (along with glucosyltransferases UGT74F1/UGT74F2) also conjugates and inactivates SA to modulate disease resistance (Huang et al., 2020a; Bauer et al., 2021), further emphasizing the regulatory and metabolic convergence of NHP and SA. Complementing these studies, recent genetic analyses demonstrated that autoimmunity and growth suppression in the *camta1/2/3* triple mutant can be reversed by mutations in the NHP biosynthetic genes *ALD1* and *FMO1* (Sun et al., 2020).

There are several major knowledge gaps regarding how NHP affects growth and development, particularly on its mechanistic impact on canonical growth hormones like auxin, GA, and brassinosteroid (BR). Although crosstalk with hormones is relatively uncharacterized, the NHP precursor Pip has been described as an osmoprotectant in both bacteria and plants (Gouesbet et al., 1994; Moulin et al., 2006; Pérez-García et al., 2019), and this could have profound consequences on overall plant physiology. Pip levels were found to increase under hyperosmotic conditions and decrease under hypo-osmotic conditions, although the authors did not measure growth phenotypes (Moulin et al., 2006). During osmotic stress, lysine-ketoglutarate reductase and saccharopine dehydrogenase can regulate L-lysine (Pip/NHP precursor) catabolism (Moulin et al., 2006). Under drought conditions, Pip accumulates in the roots/rhizosphere of sorghum, likely mediating root growth suppression (Caddell et al., 2020). Strawberry leaves with a stunted growth phenotype were also found to accumulate Pip after chilling or treatment with maleic hydrazide (Yatsu and Boynton, 1959).

Consistent with the negative impact of NHP on growth phenotypes, transcriptome analyses in *Arabidopsis* revealed that NHP-suppressed genes are associated with photosynthesis and primary metabolism, particularly those involved in fatty acid and amino acid biosynthesis (Yildiz et al., 2021). Close examination of their transcriptome data reveal that certain NHP-downregulated genes are associated with the auxin (*IAAs* and *AUXIN RESPONSE FACTORS/ARF*s), BR (*BRASSINOSTEROID INSENSITIVE 1/BRI1* and *BRI1-EMS-SUPPRESSOR 1/BES1*), and GA pathways (*DELLA, GA2OX*). It is important to highlight that NHP-downregulation of these growth/development-related genes is less pronounced than in biologically induced SAR (Yildiz et al., 2021).

In the future, it would be interesting to conduct focused mechanistic studies on how NHP intercepts various growth hormone pathways and to determine whether common molecular components are targeted by both SA and NHP. Because of the known functional synergism between SA and NHP, it is intriguing to speculate that NHP influences these other hormones through similar mechanisms perturbed by SA. It is also unclear if the antagonistic effect of NHP on growth/development is dependent on or parallel with functional SA signaling. These potential directions will establish whether NHP is central to the growth-immunity balance just like SA.

## SA and NHP at the Crossroads of Growth-Defense Homeostasis

Salicylic acid and possibly NHP can impact growth and developmental processes, sometimes directly regulating other hormone pathways. SA, in particular, has been well-demonstrated for its central role in the growth-immunity balance (Huot et al., 2014). It is not surprising that growth-related pathways (e.g., major growth hormones) can directly impinge on SA biosynthesis and signaling (Figure 2).

A well-demonstrated example is auxin signaling modulating the SA pathway (Wang et al., 2007). Lowering auxin levels *via* GH3.5 is associated with higher SA levels, contributing to this canonical plant tradeoff (Hagen and Guilfoyle, 2002). *AUXIN SIGNALING F BOX PROTEIN 1* (*AFB1*) overexpression enhances auxin signaling, resulting in lower SA levels and increased host susceptibility (Robert-Seilaniantz et al., 2011). Auxin may also negatively impact the NHP pathway. NHP biosynthetic genes *ALD1* and *FMO1* are downregulated after treatment with the auxin indole-3-acetic acid as revealed by transcriptome datasets in the *Gene Expression Atlas*.^1^ However, further mechanistic investigations are still lacking.

Another class of hormones, BRs, have differential relationships with SA depending on the species (De Vleesschauwer et al., 2012). In rice, BR treatment represses SA signaling, while the opposite is observed in *Arabidopsis* (De Vleesschauwer et al., 2012). Like auxin, BR also antagonizes SA by blocking rice resistance. Specifically, the synthetic SA analog benzothiadiazole is less effective against the root oomycete pathogen *Pythium graminicola* after BR treatment (De Vleesschauwer et al., 2012). In contrast to BRs, exogenous GA promotes expression of *ICS1* and *NPR1*, leading to increased SA levels in *Arabidopsis* (Alonso-Ramírez et al., 2009). The SA pathway is also influenced by another growth-related hormone, cytokinin (CK). The CK-associated type-B response regulator 2 (ARR2) directly interacts with TGA3 that regulates SA-responsive *PR* genes (O’Brien and Benková, 2013), thereby increasing *Arabidopsis* resistance against *Hyaloperonospora arabidopsidis* after CK treatment (Argueso et al., 2012). In rice, CK and SA synergistically activate *PR* gene expression against *Magnaporthe oryzae* infection (Jiang et al., 2013), although CK did not induce expression of SA signaling regulators *NPR1* and *WRKY45* (Jiang et al., 2010). Finally, it has been demonstrated that strigolactones can induce SA accumulation (Omoarelojie et al., 2019). How these hormones intercept NHP levels and signaling remain unclear.

Apart from major hormone pathways, the growth-defense balance can be regulated by the Target of Rapamycin (TOR) kinase (De Vleesschauwer et al., 2018). TOR is a broadly conserved eukaryotic master regulator of growth and development (Shi et al., 2018). In rice, TOR aids growth and development at the expense of immunity by antagonizing SA and suppressing PTI (De Vleesschauwer et al., 2018). Increased SA-dependent responses were observed after TOR disruption genetically or pharmacologically, while overexpressing TOR resulted in downregulated SA-associated genes (De Vleesschauwer et al., 2018). Currently, the impact of TOR on NHP biosynthesis/signaling is unknown.

These studies altogether suggest a model that growth and developmental processes mechanistically impact the SA pathway. It would be intriguing to investigate whether NHP biosynthesis and signaling are similarly impacted by major growth hormones and TOR, and whether this occurs dependently or independently of SA. It would not be surprising to discover direct functional linkage of growth/developmental processes on NHP biosynthesis and signaling, since growth suppression is associated with NHP over-accumulation (Pastorczyk-Szlenkier and Bednarek, 2021) and the NHP pathway exhibits close mechanistic connections to SA (Zeier, 2021).

## Conclusion

Increased SA and NHP levels through mutual potentiation lead to effective plant immunity against biotrophic and hemibiotrophic pathogens (Vlot et al., 2021; Zeier, 2021). Optimal defenses can sometimes result in tradeoffs to growth and development (Huot et al., 2014). Indeed, higher SA and NHP levels lead to dwarfed plants (Rivas-San Vicente and Plasencia, 2011; Cai et al., 2021). However, further studies on the broad conservation and/or specificity of SA/NHP-growth antagonism should be performed in other plant taxa. Notably, the NHP pathway and its role in SAR has been demonstrated in various plant species (Schnake et al., 2020). Although there is intensive crosstalk between SA and NHP, the impact of elevated NHP levels on plant physiology is largely unexplored. The additional dimensions of plant-microbiome and plant-environment interactions (Lebeis et al., 2015; Nazar et al., 2015; Pluhařová et al., 2019; Conesa et al., 2020) *via* the SA and NHP pathways remain low-hanging fruits, which can be facilitated by recent global datasets on microbiota assembly and hormone interactomes (Altmann et al., 2020; Trivedi et al., 2020).

Ultimately, the dream goal would be to optimize the plant’s growth-defense balance to maximize both yield and immune resilience (Mathan et al., 2016; Kim et al., 2021). Apart from tunable calibration of SA levels and signaling (van Butselaar and Van den Ackerveken, 2020), a potential avenue to bypass the growth-defense tradeoff may be optimally manipulating the NHP levels (Cai et al., 2021). Nevertheless, targeted engineering of this pathway still needs to be fully demonstrated and whether unforeseen collateral damage result from bypassing growth-defense tradeoffs must be investigated. These open questions and future directions highlight the exciting promise of elucidating and dissecting the mechanisms underpinning the equilibrium between plant growth and immunity.

## Author Contributions

CDMC conceptualized the review, supervised the research, and acquired funding. AS and VS surveyed the literature and synthesized the sources. AS, VS, and CDMC wrote the final version of the paper. All authors contributed to the article and approved the submitted version.

## Funding.

We are grateful for research funding from the Natural Sciences and Engineering Research Council of Canada (NSERC) Discovery Grant, Canada Foundation for Innovation, Ontario Research Fund, and the Faculty of Science at Wilfrid Laurier University (to CDMC). We also acknowledge funding from the Mitacs Research Training Award (to VS).

## Conflict of Interest

The authors declare that the research was conducted in the absence of any commercial or financial relationships that could be construed as a potential conflict of interest.

## Publisher’s Note

All claims expressed in this article are solely those of the authors and do not necessarily represent those of their affiliated organizations, or those of the publisher, the editors and the reviewers. Any product that may be evaluated in this article, or claim that may be made by its manufacturer, is not guaranteed or endorsed by the publisher.

## Footnotes

^1^ https://www.ebi.ac.uk/gxa/home
